# Melatonin promotes skin flap survival by inhibiting ferroptosis via activation of the Nrf2/HO-1 pathway

**DOI:** 10.1093/burnst/tkag012

**Published:** 2026-02-02

**Authors:** Mi Liu, Jiacheng Hu, Jiayi Huang, Zhefeng Cai, Peng Zou, Jing Bu, Shanshan Yu, Yuxi Zhou, Xiaoqiong Jiang, Lianfang Gan, Shuhong Tian, Lei Dong, Fenzan Wu, Huiming Deng, Jian Xiao

**Affiliations:** Affiliated Cixi Hospital, Wenzhou Medical University, No. 999 South Second Ring Road, Cixi, Zhejiang 315300, China; Oujiang Laboratory (Zhejiang Lab for Regenerative Medicine, Vision and Brain Health), School of Pharmaceutical Science, Department of Wound Healing of the First Affiliated Hospital, Wenzhou Medical University, University Town, Chashan, Wenzhou, Zhejiang 325035, China; Oujiang Laboratory (Zhejiang Lab for Regenerative Medicine, Vision and Brain Health), School of Pharmaceutical Science, Department of Wound Healing of the First Affiliated Hospital, Wenzhou Medical University, University Town, Chashan, Wenzhou, Zhejiang 325035, China; Cixi Biomedical Research Institute, Wenzhou Medical University, No. 508, East Second Ring North Road, Cixi, Zhajiang 315300, China; Oujiang Laboratory (Zhejiang Lab for Regenerative Medicine, Vision and Brain Health), School of Pharmaceutical Science, Department of Wound Healing of the First Affiliated Hospital, Wenzhou Medical University, University Town, Chashan, Wenzhou, Zhejiang 325035, China; Affiliated Cixi Hospital, Wenzhou Medical University, No. 999 South Second Ring Road, Cixi, Zhejiang 315300, China; Oujiang Laboratory (Zhejiang Lab for Regenerative Medicine, Vision and Brain Health), School of Pharmaceutical Science, Department of Wound Healing of the First Affiliated Hospital, Wenzhou Medical University, University Town, Chashan, Wenzhou, Zhejiang 325035, China; Cixi Biomedical Research Institute, Wenzhou Medical University, No. 508, East Second Ring North Road, Cixi, Zhajiang 315300, China; Oujiang Laboratory (Zhejiang Lab for Regenerative Medicine, Vision and Brain Health), School of Pharmaceutical Science, Department of Wound Healing of the First Affiliated Hospital, Wenzhou Medical University, University Town, Chashan, Wenzhou, Zhejiang 325035, China; Cixi Biomedical Research Institute, Wenzhou Medical University, No. 508, East Second Ring North Road, Cixi, Zhajiang 315300, China; Oujiang Laboratory (Zhejiang Lab for Regenerative Medicine, Vision and Brain Health), School of Pharmaceutical Science, Department of Wound Healing of the First Affiliated Hospital, Wenzhou Medical University, University Town, Chashan, Wenzhou, Zhejiang 325035, China; Cixi Biomedical Research Institute, Wenzhou Medical University, No. 508, East Second Ring North Road, Cixi, Zhajiang 315300, China; Oujiang Laboratory (Zhejiang Lab for Regenerative Medicine, Vision and Brain Health), School of Pharmaceutical Science, Department of Wound Healing of the First Affiliated Hospital, Wenzhou Medical University, University Town, Chashan, Wenzhou, Zhejiang 325035, China; Cixi Biomedical Research Institute, Wenzhou Medical University, No. 508, East Second Ring North Road, Cixi, Zhajiang 315300, China; Oujiang Laboratory (Zhejiang Lab for Regenerative Medicine, Vision and Brain Health), School of Pharmaceutical Science, Department of Wound Healing of the First Affiliated Hospital, Wenzhou Medical University, University Town, Chashan, Wenzhou, Zhejiang 325035, China; Hainan Pharmaceutical Research and Development Science Park, Hainan Medical University, No. 3 Xueyuan Road, Haikou, Hainan 571199, China; Hainan Pharmaceutical Research and Development Science Park, Hainan Medical University, No. 3 Xueyuan Road, Haikou, Hainan 571199, China; Cixi Biomedical Research Institute, Wenzhou Medical University, No. 508, East Second Ring North Road, Cixi, Zhajiang 315300, China; Affiliated Cixi Hospital, Wenzhou Medical University, No. 999 South Second Ring Road, Cixi, Zhejiang 315300, China; Oujiang Laboratory (Zhejiang Lab for Regenerative Medicine, Vision and Brain Health), School of Pharmaceutical Science, Department of Wound Healing of the First Affiliated Hospital, Wenzhou Medical University, University Town, Chashan, Wenzhou, Zhejiang 325035, China; Oujiang Laboratory (Zhejiang Lab for Regenerative Medicine, Vision and Brain Health), School of Pharmaceutical Science, Department of Wound Healing of the First Affiliated Hospital, Wenzhou Medical University, University Town, Chashan, Wenzhou, Zhejiang 325035, China

**Keywords:** Melatonin, Skin flap, Ferroptosis, Oxidative stress

## Abstract

**Background:**

Random skin flap application is considerably limited by postoperative complications, particularly distal tissue ischemia and necrosis. Melatonin, a molecule with well-documented antioxidant and cytoprotective properties, has shown promise in protecting ischemic tissues. However, its specific role in regulating ferroptosis during ischemic flap injury, as well as its safety and efficacy in primate models (a key step for clinical translation), remains to be systematically validated. In this study, we aimed to promote angiogenesis within flap tissue through exogenous melatonin administration and to inhibit ferroptosis to mitigate ischemia–reperfusion injury, presenting a novel strategy for enhancing flap survival rates.

**Methods:**

A random skin flap was constructed in C57BL/6 J mice. After melatonin treatment for seven days, the influence of melatonin on the levels of oxidative stress, iron accumulation, and mitochondrial morphology within the skin flap tissue was assessed. We used Transwell migration assays, tube formation assays, flow cytometry, and immunofluorescence staining to determine the effects of melatonin *in vitro*. The ferroptosis inducer erastin was used in combination with melatonin to treat random skin flap mice and *tert*-butyl hydroperoxide (TBHP)–induced cellular models, and the pathway through which melatonin counteracts iron mutations was explored. Lastly, we conducted experiments using nonhuman primate models and analyzed the protective effects of melatonin on ischemic flaps in macaques, highlighting its potential for clinical translation.

**Results:**

Melatonin ameliorated the survival area of ischemic flaps in mice, enhanced angiogenesis, reduced mitochondrial damage, and also suppressed lipid peroxidation and iron ion accumulation. Melatonin attenuated TBHP-induced cell death, lipid peroxidation, and mitochondrial damage *in vitro*. Further mechanistic studies revealed that melatonin inhibited ferroptosis, accompanied by nuclear translocation of nuclear factor E2-related factor 2 (Nrf2), and increases the expression of downstream gene (effector) heme oxygenase-1 (HO-1). More importantly, experiments in macaques demonstrated that melatonin could enhance flap viability and angiogenesis, and exhibited a good safety profile.

**Conclusion:**

Melatonin enhanced flap viability in mice and macaques by inhibiting ferroptosis, boosting angiogenesis, and attenuating oxidative stress injury.

## Highlights

- Melatonin significantly improved the survival rate of random-pattern skin flaps in both mouse and nonhuman primate models.
- Melatonin promoted tissue regeneration and enhanced flap survival by inhibiting ferroptosis, enhancing angiogenesis, and reducing oxidative stress.
- Nrf2/HO-1 signaling was involved in melatonin-mediated ferroptosis in skin flaps and *tert*-butyl hydroperoxide–induced injury in human umbilical vein endothelial cells.
- The therapeutic targeting of ferroptosis with melatonin offers a novel strategy for preventing flap necrosis.

## Background

Given that these flaps demonstrate nondependence on specific axial vessels, the random skin flaps serve as a versatile solution in clinical tissue reconstruction [1, 2]. Moreover, the blood supply is derived from a random subcutaneous vascular network, facilitating flexibility in placement and design without strict anatomical constraints [3, 4]. However, this adaptability allows them to be suitable for repairing soft tissue and skin defects resulting from diverse etiologies [5]. Nevertheless, tissue repair represents a highly complex and dynamic process [6]. Flaps exceeding a 2:1 length-to-width ratio commonly exhibit limited distal perfusion due to the intrinsic limitations of random vascularization [7, 8]. In light of the evidence that hypoperfusion disrupts local homeostasis, induces apoptosis, and may eventually result in ischemic necrosis, improving flap perfusion remains critical challenge in reconstructive surgery. Despite continuous advances in surgical techniques, necrosis rates of random skin flaps are still reported to range between 10% and 15%, highlighting the need for more effective interventions [8, 9]. Therefore, developing novel strategies to increase flap survival represents a significant clinical priority.

While many studies have investigated various drugs and biomaterials to improve flap survival rates, clinical outcomes remain suboptimal [10, 11]. Melatonin, primarily recognized for its role in treating circadian rhythm disorders, has recently shown promise in dermatological applications [12, 13]. Research revealed that melatonin could effectively reduce oxidative stress across different pathological conditions [14]. For instance, it has been shown to alleviate oxidative stress and DNA injury, protecting mouse testes from palmitate-induced lipotoxicity [15]. Given that oxidative stress caused by ischemia–reperfusion injury is a key factor in poor flap survival [16, 17], and earlier studies have demonstrated that melatonin’s protective effects against necrosis in random-pattern flaps in rats [18]. We hypothesized that melatonin may improve flap survival by promoting angiogenesis and reducing oxidative stress.

Ischemia–reperfusion injury in flap tissue is a significant challenge in surgical procedures, arising from the abrupt halt and subsequent resumption of blood flow following. This process triggers oxidative stress, causing tissue damage and diminishing survival rates [17]. Ferroptosis, a distinct type of ferrodependent cell death, is marked by the aggregation of lipid peroxides [19]. Here, free iron ions drive the production of reactive oxygen species (ROS) through the Fenton reaction, creating a vicious cycle that intensifies oxidative damage to cell membranes [20]. This damage reduces membrane flexibility, increases its permeability, and ultimately causes widespread cell death. Previous research has linked ferroptosis to the development of ischemia–reperfusion, highlighting its close connection to oxidative stress [21–23]. For example, blocking ferroptosis has been shown to reduce oxidative stress and ease injury in myocardial ischemia–reperfusion models [24]. Recent findings suggested that melatonin is a potential inhibitor of ferroptosis [14]. its impact in random flap models remains uncharted territory.

In this study, we explored whether melatonin could boost flap survival by examining its impact on angiogenesis, oxidative stress, and ferroptosis. We further elucidated the mechanisms through which melatonin improved flap viability in a nonhuman primate (NHP) ischemic flap model. Our results demonstrated that melatonin enhanced tissue regeneration and flap survival by inhibiting ferroptosis, promoting angiogenesis, and reducing oxidative stress, offering theoretical support and new therapeutic avenues for improving flap survival clinically.

## Methods

### Animals and ethics approval

C57BL/6 J mice (6-week-old males) were sourced from Cyagen Biosciences in Suzhou, China. These mice were kept in a specific pathogen-free animal care facility, enjoying a 12-h light/dark cycle, steady temperature of 22 ± 1°C, and 40%–60% air humidity. All experimental protocols received approval from Wenzhou Medical University’s Animal Care and Use Committee (wydw2024-0322).

Six cynomolgus macaques, aged between 3 and 5 years, were obtained from Hainan Xinzhengyuan Biotechnology. The macaques were housed at the Hainan Drug Safety Evaluation Research Center. All macaque procedures were approved by the center’s Animal Care and Use Committee [20220915-NHP (01)].

### Random skin flap model and groups

For mouse studies, random flaps were created using a previously reported method. In brief, the mice were anesthetized and their dorsal hair removed; a 1.5 cm × 4.5 cm incision was performed in the dorsal area. The lateral sacral arteries were excised bilaterally, and the flap was subsequently sutured using surgical sutures. The flap was subdivided into three equal regions: the proximal region (area I), the middle region (area II), and the distal region (area III). The mice were assigned randomly into four groups: saline control group (Ctrl, n = 6), melatonin group (Mel, 10 mg/kg, *n* = 6; MedChemExpress, HY-B0075), erastin group (Era, 10 mg/kg, *n* = 6; MedChemExpress, HY-15763), Era + Mel group (*n* = 6), and normal skin as a negative control.

The NHP flap model was anaesthetized via intravenous injection of atropine (0.1 mg/kg) combined with 3% pentobarbital sodium intravenously. Following depilation, a 3 cm × 9 cm incision was made on the animal’s back. Six macaques were split into two groups: saline control group (Ctrl) and melatonin-treated group (Mel, 10 mg/kg). After surgery, the melatonin treatment group was administered daily by oral gavage for 7 days postoperation, while the control group received an equivalent saline volume. Tissues were harvested and analyzed on day 7.

### Observation and assessment of the flap

The appearance, tissue elasticity, and necrosis of the dorsal skin flaps in mice and macaques were observed at the indicated times. The flaps were photographed and analyzed by digital imaging on the third and seventh days after surgery, and a black skin flap with a hard texture and poor elasticity was considered necrotic. The flap viable area was measured using ImageJ, and the survival rate was then calculated as (viable area/gross area) × 100%.

### Laser Doppler imaging

On day 7 postoperation, laser Doppler blood flow (LDBF) microscopy (Moor Instruments, Axminster, UK) was used to measure blood flow to the mouse flap. Blood flow perfusion (PU) was evaluated using moorLDI review software version 6.1.

### Histology and immunostaining analysis

Skin tissue was fixed in paraffin and optimal cutting temperature compound (OCT), and stored at room temperature and − 80°C, respectively. The paraffin-embedded skin sections (5 μm) were deparaffinized, rehydrated, and subjected to H&E staining (Solarbio, G1121) and Prussian blue iron staining (Solarbio, G1429). Images were taken using a Nikon ECLIPSE Ni-U microscope (Japan).

For terminal dUTP nick end labeling (TUNEL) staining, paraffin-embedded skin sections underwent the TUNEL assay (Beyotime, C1086) following instructions. Images were captured with a Nikon AX confocal microscope (Japan).

For dihydroethidium (DHE) staining, frozen skin sections (15 μm) were incubated with DHE (5 μmol/L; Beyotime, S0063) for 30 min at 37°C in a dark chamber. Images were captured with a Nikon AX confocal microscope (Japan).

For immunofluorescence staining, paraffinized skin sections were deparaffinized and rehydrated, and the sections were fixed for 15 min in Tris-EDTA buffer (pH 9.0) at 95°C, followed by a 60-min incubation in blocking buffer at room temperature. Afterward, the primary antibody was applied and incubated at 4°C for 12 h. After three phosphate buffered saline (PBS) washes, the sections were treated with the secondary antibody at room temperature for 60 min. Nuclei were then stained with 4’,6-diamidino-2-phenylindole (DAPI. Primary antibodies utilized were as follows: anti-CD31 (rabbit, 1:2000; Abcam, ab182981), anti-E-cadherin (rat, 1:200; Abcam, ab282277; rabbit, 1:100; ThermoFisher, 36-1900), anti-MMP9 (matrix metallopeptidase 9; rabbit, 1:50; Abcam, ab283575), anti-F4/80 (rabbit, 1:400; HaoKe, B06001R), anti-CD206 (rabbit, 1:200; HaoKe, B03006R), anti-Nrf2 (rabbit, 1:200; Proteintech, 16396-1), and anti-4-HNE (mouse, 1:100; Abcam ab48506). The secondary antibodies (1:400) were conjugated to Alexa Fluor 488 or 594 (Abcam). Images were taken with a Nikon AX confocal microscope (Japan).

### Cell culture and treatment

Human umbilical vein endothelial cells (HUVECs) were acquired via the China Academy of Sciences Cell Bank (Shanghai, China). The cells were cultured in medium containing 10% (v/v) fetal bovine serum (Gibco, USA) at 37°C with 5% CO_2_. To comprehensively evaluate ferroptosis *in vitro*, we employed two distinct inducers: *tert*-butyl hydroperoxide (TBHP), a direct oxidant that causes lipid peroxidation, and erastin, a specific inhibitor of system Xc^−^ that disrupts glutathione-dependent antioxidant defense [25–27]. HUVECs were treated with different concentrations of TBHP (0, 20, 40, 60, 80, and 100 μM) to determine the optimal concentration. The cells were subsequently divided into these conditions: control-treated group (Ctrl), TBHP-treated group (cells exposed to 40 μM TBHP), TBHP + Mel-treated group (cells exposed simultaneously to 40 μM TBHP and 20 μM Mel simultaneously), TBHP + Era-treated group (cells exposed simultaneously to 40 μM TBHP and 10 μM Era simultaneously), and TBHP + Era + Mel-treated group (cells exposed simultaneously to 40 μM TBHP, 10 μM Era and 20 μM Mel simultaneously).

### Cell viability analysis

The cell counting kit-8 (CCK-8) assay was employed to detect HUVECs’ viability under various treatments. In short, HUVECs were seeded into 96-well plates (6 × 10^3^ cells/100 μl) and cultured for 12 or 24 h. After the indicated treatments were performed, the fresh medium with 10% CCK-8 (Beyotime, C0041) was added and then incubated further at 37°C for 2 h. The cell viability was quantified through absorbance at 450 nm.

### Cell proliferation analysis

For cell proliferative analysis, 5-ethynyl-2′-deoxyuridine (EdU) labelling was carried out according to the manufacturer’s guide (Beyotime, C0078S), then, nuclei were stained with DAPI for 5 min. Images were captured with a Nikon AX confocal microscope (Japan).

### Transwell analysis

HUVEC migration was assessed using Transwell chambers (Corning, 3398). The treated cells were cultured for 24 h in the upper chamber, which contained 400 μl of serum-free medium at 37°C. Migrated cells in the lower chamber were fixed in 4% formaldehyde and then stained using crystal violet (Beyotime, C0121). Three random fields per chamber were examined and averaged under a multifunctional microscope (Olympus, APEXVIEW APX100, Japan).

### Tube formation analysis

HUVECs were inoculated into a 24-well plate coated with Matrigel (1 × 10^5^ cells/ml) and cultured at 37°C for 6 h, to allow tube formation. The cells were then stained for 30 min in Calcein AM (Beyotime, C2012). Images were captured with a Nikon AX confocal microscope (Japan). ImageJ was employed to quantify the connections and overall branch length.

### Lipid peroxidase analysis

Lipid peroxidase contents were measured with a BODIPY 581/591 C11 kit (Invitrogen, D3861). HUVECs were seeded into confocal microplates (1 × 10^5^ cells/well) and cultured for 24 h. A C11-BODIPY probe was administered to the cells (2 μM), which were then incubated for 5 min at 37°C. Confocal microscope (Nikon AX, Japan) visualized C11-unoxidized stage (red) and the C11-oxidized stage (green), with analysis performed using ImageJ 6.0.

### Reactive oxygen species assay

The content of ROS was detected utilizing 2,7-dichlorodihydro fluorescein diacetate (DCFH-DA,Beyotime, S0034S) staining. The HUVECs underwent staining with 10 μM DCFH-DA at 37°C for 20 min. After trypsin digestion to obtain a single-cell suspension, cells were resuspended in medium supplemented with 5% fetal bovine serum (FBS). The fluorescence was analyzed using CytoFLEX S (Beckman Coulter, USA), with outcomes analyzed via FlowJo 10.8.1.

### Iron detection

HUVECs were seeded into confocal dishes (1 × 10^5^ cells/well) and treated for 24 h. Afterward, the cells were then incubated with FerroOrange (1 μM; Dojindo Molecular Technology, F374) for 30 min at 37°C in the dark to detect intracellular ferrous ions. After three 5-min PBS washes, images were scanned with a Nikon AX confocal microscope (Japan), and the integrated density was determined using ImageJ 6.0.

Moreover, total and ferrous iron content in cells and flap tissues were determined using iron assay kits (Elabscience, E-BC-K772-M and E-BC-K773-M). Briefly, cells and tissues were lysed in iron solution, centrifuged at 12 000  g for 10 min, and the supernatant was mixed with iron probes and incubated additionally at 37°C for 30 min. Absorbance was recorded at 593 nm.

### Transmission electron microscopy

Using ultrastructure observation with transmission electron microscopy (TEM), the skin tissues and HUVECs were collected at the indicated times and then fixed for 24 and 2 h, respectively, in 2.5% glutaraldehyde (pH 7.4). Following three PBS washes, all the samples were further immersed in osmium tetroxide at 4°C for 2 h, then dried and embedded in Epon–Araldite resin for ultrathin sectioning. Sections were stained with uranyl acetate-lead citrate and examined via JEM-F200 electron microscopy (Japan).

### Oxidative stress test

Cell or flap tissues were collected in centrifuge tubes. The concentrations of superoxide dismutase (SOD), malondialdehyde (MDA), glutathione (GSH), glutathione peroxidase (GSH-PX), and oxidized glutathione (GSSG) were detected using the corresponding commercial assay kits, following the protocols.

### Quantitative reverse-transcription polymerase chain reaction

First, total RNA was harvested from flap samples or HUVECs employing the RNA-Easy isolation reagent kit (Vazyme, R701). Complementary deoxyribonucleic acid (cDNA) was synthesized using PrimeScript RT Master Mix (Takara, RR036Q). The primer sequences are detailed in Table S1.

### Western blot

Flap tissues and cells were lysed in radio-immunoprecipitation assay lysis (RIPA) buffer (Beyotime, P0013B) incorporating 1 mM phenylmethylsulfonyl fluoride (PMSF, Beyotime, ST505) and centrifuged at 12 000 rpm at 4°C for 20 min. After centrifugation, the supernatant was collected and resolved by sodium dodecyl sulfate-polyacrylamide gel electrophoresis (SDS-PAGE, Biosharp, BL502B) and transferred to a polyvinylidene fluoride (PVDF) membrane (Millipore, IPVH00010). The membranes were blocked for 1 h at room temperature with 5% skim milk, then incubated with the designated primary antibody at 4°C for 12 h. Following tris-buffered saline with tween-20 (TBST) washing, the membrane was incubated with secondary antibodies labeled horseradish peroxidase (HRP) and gently shaken for 1 h. Following further TBST washes, the membrane was visualized using an enhanced chemiluminescence (ECL) detection reagent (Bio-Rad, 1705061) and imaged via a ChemiDoc image capture system (Bio-Rad, USA). Gray values were measured using ImageJ 6.0.

The primary antibodies utilized used were as follows: solute carrier family 7 member 11 (SCL7A11; rabbit, 1:1000; Abcam, ab175186), glutathione peroxidase 4 (GPX4; rabbit, 1:1000; Abcam, ab125066), HO-1 (mouse, 1:1000; Proteintech, 66743), Nrf2 (rabbit, 1:1000; Proteintech, 16396-1), and GAPDH (glyceraldehyde-3-phosphate dehydrogenase; rabbit; 1:10 000, Abcam, ab181603). The secondary antibodies (1:10 000) were goat anti-rabbit HRP-conjugated (Biasharp, BL003A) or goat anti-mouse HRP-conjugated (Biasharp, BL001A).

### Statistical analysis

Data are shown as mean ± SEM and analyzed utilizing GraphPad Prism 8.0. Statistical comparisons were conducted using Student’s *t*-tests, Kruskal–Wallis H tests, and one-way analysis of variance (ANOVA). Differences were considered statistically significant at *P* < 0.05. All treatments were administered in triplicate in three independent experiments.

## Results

### Melatonin alleviates random skin flap necrosis and *tert*-butyl hydroperoxide–induced injury in human umbilical vein endothelial cells

**Figure 1:**
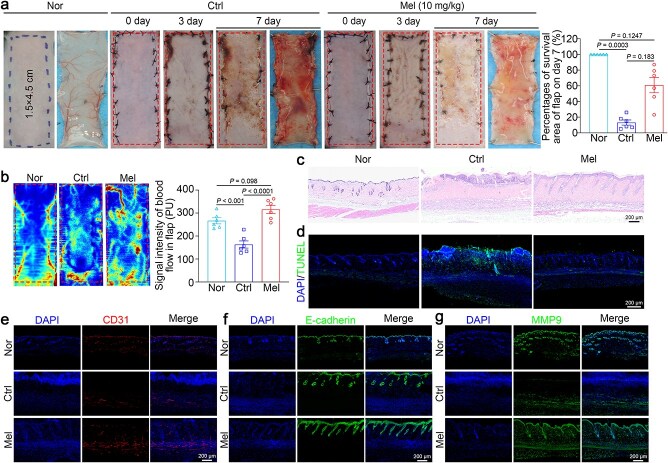
Melatonin enhances angiogenesis and alleviates necrosis in random skin flaps. A murine random flap model was developed. The experimental group received exogenous melatonin (Mel) postoperatively, while the flap control group received an equivalent volume of saline, normal skin (Nor) from untreated mice as a negative control. Animals were euthanized on postoperative day 7 to assess the flap survival area and subcutaneous microcirculation. (**a**) Gross appearance of the flaps on days 0, 3, and 7 postsurgery, and quantification of the survival area on day 7 (*n* = 6 per group). (**b**) Subcutaneous vascular network and blood flow signal intensity within the flap as shown by LDBF imaging (*n* = 6 per group). (**c**) Representative images of H&E-stained sections from normal, saline-treated, or melatonin-treated flaps. Scale bar: 200 μm. (**d**) Representative images of TUNEL staining indicating cell death in skin flap tissue treated with normal, saline, or melatonin. Scale bar: 200 μm. (**e**–**g**) Immunofluorescence staining for the vascular markers CD31 (red) and E-cadherin (green) and the proangiogenic factor MMP9 (green) in area II skin flaps. Scale bar: 200 μm. The data are shown as the means ± SEM, the results of the Kruskal–Wallis H analysis (a) and one-way ANOVA (b) with Tukey’s multiple comparisons test are shown. *Nor* normal, *Ctrl* control, *Mel* melatonin, *TUNEL* terminal dUTP nick end labeling, *MMP9* matrix metallopeptidase 9

To explore the benefits of melatonin on the survival of random skin flaps, we developed a skin flap model on the dorsal skin of mice and divided them randomly into two groups. Melatonin (Mel, 10 mg/kg) suspended in saline was administered orally for seven consecutive days immediately after flap surgery, the flap control group administered the equivalent volume of saline (Ctrl), normal skin was used as a negative control (Nor). We first observed the morphology and histological invasion. By postoperative day 3, the flaps in the two groups showed edema, without obvious necrosis in area III (Fig. 1a). Tissue necrosis was initially observed in region III. On postoperative day 7, melatonin-treated skin necrosis exhibited dramatic improvement. Laser Doppler flowmetry examination was employed to evaluate percutaneous vascular networks in flaps; the melatonin group exhibited increased blood flow signals in ischemic flaps compared with the control group (Fig. 1b).

Furthermore, area II flap samples were harvested and analyzed on day 7. Histological examination revealed that the flap exhibited excellent healing and structural integrity after melatonin treatment, with increased microvascular density (Fig. 1c). TUNEL staining was employed to evaluate cell death in the flap tissues. The results revealed a notable rise in TUNEL-positive cells in the necrotic zones of untreated flaps, while melatonin treatment markedly reduced their numbers (Fig. 1d). These findings collectively confirmed that melatonin helps minimize tissue injury and cell death in skin flaps.

Immunofluorescence staining confirmed elevated CD31 expression in flaps treated with melatonin (Fig. 1e). As CD31 is expressed in both endothelial cells and macrophages, we additionally conducted costaining with macrophage markers F4/80 (macrophage marker) and CD206 (M2-macrophage marker) [28, 29]. The results revealed minimal overlap between CD31 and these macrophage markers, indicating that the rise in CD31 expression was mainly due to endothelial cells (Fig. S1a). We also noted an increase in M2 macrophages in the melatonin-treated group, which contributed to tissue repair (Fig. S1b). Additionally, we investigated changes in E-cadherin and MMP9 expression, key factors in improving flap survival, reducing ischemic cell death, and supporting angiogenesis and blood supply [30, 31]. Staining of the skin tissue samples confirmed that both E-cadherin and MMP9 levels increased after melatonin treatment (Fig. 1f and g). Together, our results suggested that melatonin enhanced angiogenesis and promoted the survival of ischemic flaps.

**Figure 2:**
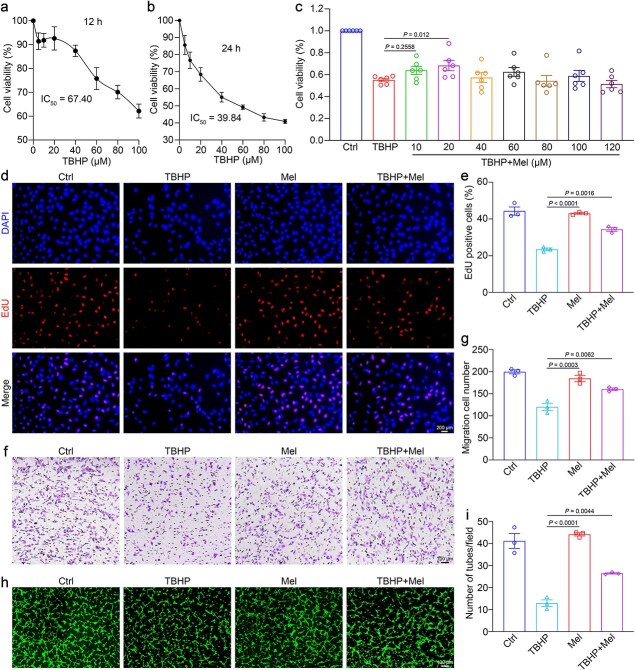
Melatonin attenuates TBHP-induced injury in HUVECs. (**a**, **b**) Cell viability was assessed using the CCK-8 assay following treatment with different concentrations of TBHP for 12 or 24 h. (**c**) CCK-8 assay to evaluate the optimally effective dose of melatonin for enhancing the survival of TBHP-HUVEs. (**d**, **e**) Proliferation of HUVECs after melatonin therapy and the number of proliferating endothelial cells. Scale bar: 200 μm. (**f**, **g**) Migratory ability of HUVECs after melatonin exposure and quantification of the number of migrated cells. Scale bar: 100 μm. (**h**, **i**) Angiogenic capacity of endothelial cells after melatonin treatment and quantification of the number of tubes formed. Scale bar: 100 μm. The data are presented as the means ± SEM, two-way ANOVA (a–c) and one-way ANOVA (e, g, i) with Tukey’s multiple comparisons test. *TBHP tert*-butyl hydroperoxide, *Ctrl* control, *Mel* melatonin, *HUVECs* human umbilical vein endothelial cells, *CCK8* cell counting kit-8, *EdU* 5-ethynyl-2′-deoxyuridine

Oxidative stress plays a pivotal role in flap necrosis and ischemia [17]. To replicate flap conditions *in vitro*, we used TBHP-HUVECs due to their ability to release oxidative stress steadily [32]. To evaluate the ideal TBHP dose for inducing oxidative stress, we exposed HUVECs to varying dosages of TBHP and assessed cellular viability using CCK-8 assays. The IC_50_ values for TBHP were 67.4 μM at 12 h and 39.8 μM at 24 h, respectively (Fig. 2a and b). We subsequently treated HUVECs with TBHP (40 μM) and different concentrations of melatonin. Our results showed that 20 μM melatonin offered promising protection on HUVECs without adverse effects (Fig. 2c). Interestingly, higher melatonin did not boost protection on HUVECs, possibly because of melatonin’s dose-dependent effects in endothelial cells, where excessive amounts could increase its toxicity.

The uncontrolled proliferation and migration of endothelial cells can severely disrupt blood vessel formation [33]. We employed EdU proliferation and transwell migration assays to assess melatonin’s ability to repair TBHP-induced damage. Notably, melatonin remarkably improved the proliferation and migration of TBHP-induced HUVECs compared to control (Fig. 2d–g). We also observed a marked rise in tube formation, suggesting vascular neogenesis, proving melatonin’s efficacy (*P* = .0044; Fig. 2h and i).

### Melatonin improves the survival of skin flaps and alleviates *tert*-butyl hydroperoxide–induced injury in human umbilical vein endothelial cells by inhibiting ferroptosis

**Figure 3:**
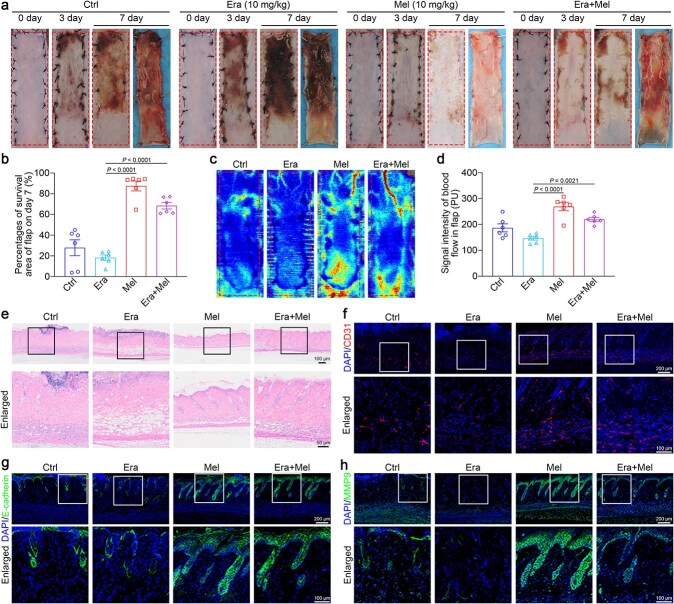
Melatonin enhances the survival of skin flaps by inhibiting ferroptosis. A murine flap wound was generated, and the mice were administered with the ferroptosis inducer erastin (Era) and/or melatonin. (**a**) Gross appearance of flaps on days 0, 3, and 7 after treatment with saline, Era, or Era + melatonin. (**b**) Quantification of the flap survival area (*n* = 6 per group). (**c**) Imaging of the subcutaneous microvascular and blood flow signal using LDBF (*n* = 6 per group). (**d**) Quantification of blood flow signal intensity. (**e**) Representative H&E images of tissues treated with saline, Era, or Era + melatonin. (**f**–**h**) Representative immunofluorescence staining for CD31 (red), E-cadherin (green), and MMP9 (green) in skin flaps. Scale bar: 200 μm; 100 μm. The data are presented as the means ± SEM, one-way ANOVA (b, d) with Tukey’s multiple comparisons test. *Ctrl* control, *Era* erastin, *Mel* melatonin, *H&E* hematoxylin and eosin, *LDBF* laser Doppler blood flow, *MMP9* matrix metallopeptidase 9

Ferroptosis, a recently identified form of cell death, arises from iron-related lipid peroxidation accumulation [20]. To investigate the potential association between ischemic flap necrosis and ferroptosis, we analyzed ischemic flap survival after treatment with the ferroptosis inducer erastin [34]. The mel-group served as the therapeutic control group. As shown in Figure 3a and b, erastin induced necrosis throughout almost all of area I on postoperative day 7, whereas the addition of melatonin effectively mitigated flap necrosis and improved flap survival. Compared with erastin treatment, melatonin treatment consistently increased the blood flow signal of ischemic flaps in mice (Fig. 3c and d). Histological staining confirmed these effects, showing widespread tissue swelling and inflammatory cell infiltration in both control and erastin groups, but melatonin clearly alleviated erastin-induced necrosis (Fig. 3e). Immunofluorescence imaging revealed strong CD31-, E-cadherin-, and MMP9-positive signals (angiogenesis-related proteins) in the melatonin group, while these signals were nearly absent in the erastin group (Fig. 3f–h). These findings suggested that melatonin contributed to new blood vessel formation and tissue repair in erastin-induced necrotic flaps.

**Figure 4:**
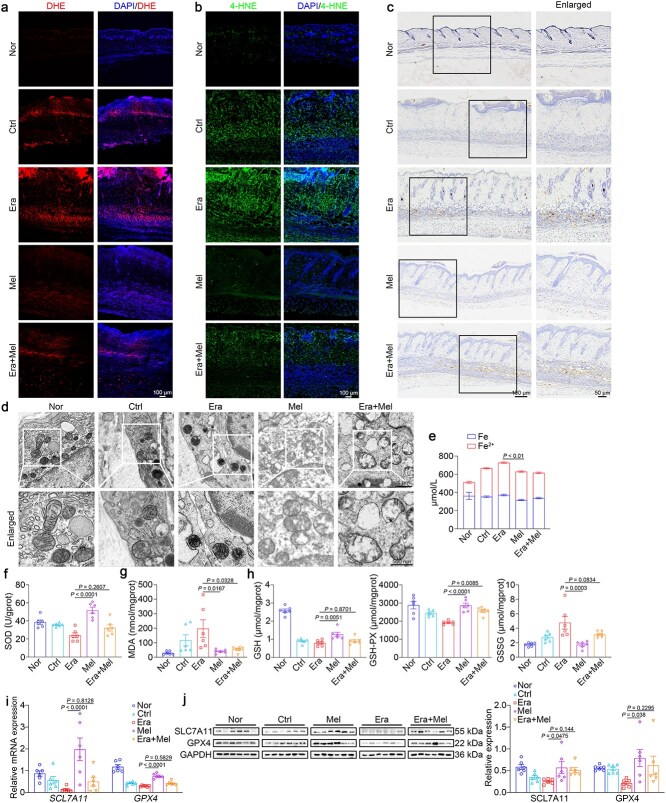
Melatonin reverses Era-induced ferroptosis. (**a**) DHE (red) staining for superoxide anion. Scale bar: 100 μm.(**b**) Immunofluorescence staining for 4-HNE (green). Scale bar: 100 μm.(**c**) Representative images of flaps administered with saline, melatonin, Era + melatonin, and normal skin using Prussian blue staining. Scale bar: 100 μm; 50 μm.(**d**) Ultrastructural changes observed by TEM. Scale bar: 1 μm; 500 nm.(**e**) Quantification of iron and Fe^2+^ levels in flap tissues (*n* = 6 per group). (**f**–**h**) Quantification of the endogenous antioxidants SOD, MDA, GSH, GSH-PX, and GSSG (*n* = 6 per group). (**i**) Analysis of *SLC7A11* and *GPX4* mRNA expression using qRT-PCR (*n* = 6 per group). (**j**) Western blot analysis of SLC7A11 and GPX4 protein expression in flap tissues (*n* = 6 per group). The data are presented as the means ± SEM, one-way ANOVA (e–j) with Tukey’s multiple comparisons test. *Nor* normal, *Ctrl* control, *Era* erastin, *Mel* melatonin, *DHE* dihydroethidium, *4-HNE* 4-hydroxynonenal, *TEM* transmission electron microscopy, *SOD* superoxide dismutase, *MDA* malondialdehyde, *GSH* glutathione, *GSH-PX*,glutathione peroxidase, *GSSG* oxidized glutathione; *SCL7A11* solute carrier family 7 member 11, *GPX4* glutathione peroxidase 4, *GAPDH* glyceraldehyde-3-phosphate dehydrogenase

Ferroptosis is characterized by enhanced ROS production, pronounced mitochondrial shrinkage, and increased membrane density [29, 30]. To investigate its impact on ischemic flap, we examined lipid peroxidation and iron accumulation in flap tissue on day 7 after surgery. Oxidative stress was evaluated using DHE staining and 4-HNE staining [31, 32]. Following melatonin treatment, ROS and 4-HNE levels in necrotic flaps markedly decreased. Notably, erastin induced more severe oxidative stress compared to the control, while melatonin administration significantly reduced ROS and lipid peroxide levels (Fig. 4a and b). Subsequently, we explored ferrous deposition and mitochondrial morphology in skin tissues from both normal and ischemic flap mice. Prussian blue staining demonstrated that melatonin decreased the iron ion content in erastin-induced severely necrotic flaps (Fig. 4c). TEM analysis showed fewer mitochondria with reduced cristae in the erastin group compared to the control, while melatonin treatment significantly ameliorated erastin-induced mitochondrial damage (Fig. 4d). Quantitative tissue iron analysis indicated that melatonin markedly reduced ferrous iron accumulation caused by erastin (Fig. 4e). We also measured antioxidant enzyme SOD, lipid-related product MDA, and antioxidants GSH and GSH-PX expression in tissue from normal and ischemic flap skin, which are crucial for maintaining the oxidative–reductive balance [35, 36]. As expected, melatonin alleviated erastin-induced oxidative stress damage (Fig. 4f–h). Furthermore, we evaluated messenger RNA (mRNA) and protein expression of SCL7A11 and GPX4, key ferroptosis-related genes [21, 31]. qRT-PCR and western blotting showed that both SCL7A11 and GPX4 mRNA and protein were noticeably greater in the Era + Mel group than in the erastin group (Fig. 4i and j). In summary, these findings indicated that ferroptosis likely occurred in ischemic flaps. Using erastin as a control, we demonstrated that melatonin enhanced ischemic flap survival and mitigated erastin-induced injury, indicating that its therapeutic effect on ischemic flaps is associated with ferroptosis inhibition.

**Figure 5:**
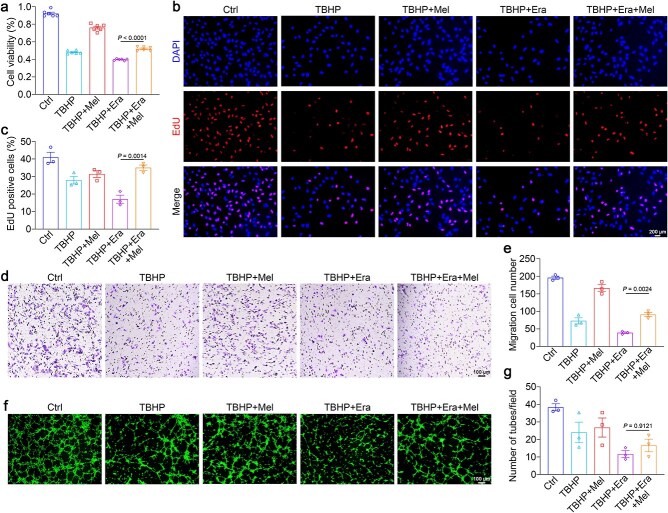
Melatonin restores the angiogenic function of TBHP + Era-induced HUVECs. In the endothelial cell oxidative stress injury model, cells were incubated with the TBHP, TBHP + Mel, TBHP + Era, or TBHP + Era + Mel. (**a**) Assaying the viability of HUVECs exposed to TBHP, TBHP + Mel, TBHP + Era, or TBHP + Era + melatonin using the CCK8 assay. (**b**, **c**) EdU staining analysis for endothelial cell proliferation and quantification of proliferating cells. Scale bar: 200 μm. (**d**, **e**) Angiogenic capacity of HUVECs and quantification of the number of tubes formed. Scale bar: 100 μm. (**f**, **g**) Migratory ability of endothelial cells and quantification of the number of migrated cells. Scale bar: 100 μm. The data are shown as the means ± SEM, one-way ANOVA (a, b, e, g) with Tukey’s multiple comparisons test. *TBHP tert*-butyl hydroperoxide, *Ctrl* control, *Era* erastin, *Mel* melatonin, *HUVECs* human umbilical vein endothelial cells, *EdU* 5-ethynyl-2′-deoxyuridine, *CCK8* cell counting kit-8

Endothelial cells are essential for restoring blood flow, controlling inflammation, and preserving flap integrity [37]. Building on the previous findings, we used TBHP and erastin (TBHP + Era) to induce ferroptosis *in vitro*, aiming to assess ferroptosis’s role in melatonin-mediated reversal of oxidative damage in TBHP-HUVECs. We first analyzed cellular viability and proliferation using CCK-8 and EdU assays; the data revealed that melatonin improved the viability and proliferation of HUVECs suppressed by both TBHP and erastin (Fig. 5a–c). Moreover, melatonin enhanced the migration and tubule-forming abilities of endothelial cells in the TBHP + Era group (Fig. 5d–g). These results indicated that melatonin exerts a specific inhibitory function on ferroptosis.

**Figure 6:**
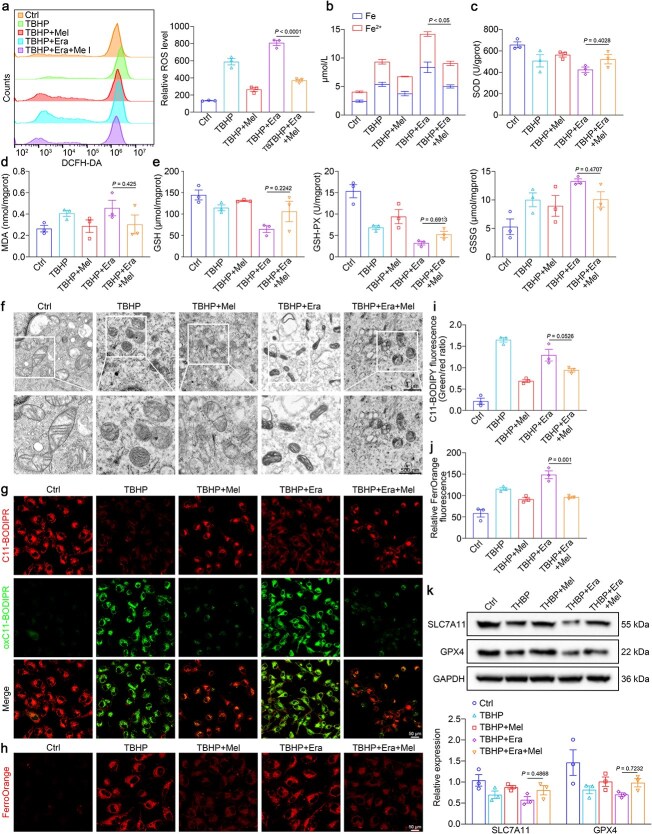
Melatonin inhibits ferroptosis and reverses the viability of TBHP + Era-induced HUVECs. (**a**) Flow cytometric analysis of ROS levels using DCFH-DA. Scale bar: 1 μm; 500 nm. (**b**) Quantification of Fe and Fe^2+^ levels in HUVECs. (**c**–**e**) Quantification of the levels of SOD, MDA, GSH, GSH-PX, and GSSG within HUVECs. (**f**) Ultrastructural changes observed by TEM. (**g**) Immunofluorescence staining with a C11-BODIPY probe for lipid peroxidation. Scale bar: 50 μm.(**h**) Immunofluorescence staining with a FerroOrange probe for Fe^2+^. Scale bar: 50 μm. (**i**) Quantification of C11-BODIPY fluorescence intensity. (**j**) Quantification of FerroOrange fluorescence intensity. (**k**) SLC7A11 and GPX4 protein expression analyzed using western blot. The data are shown as the means ± SEM, one-way ANOVA (a–e, i–k) with Tukey’s multiple comparisons test. *TBHP tert*-butyl hydroperoxide, *Ctrl* control, *Era* erastin, *Mel* melatonin, *HUVECs* human umbilical vein endothelial cells, *ROS* reactive oxygen species, *DCFH-DA* 2′,7′-dichlorofluorescin diacetate, *SOD* superoxide dismutase, *MDA* malondialdehyde, *GSH* glutathione, *GSH-PX* glutathione peroxidase, *GSSG* oxidized glutathione, *TEM* transmission electron microscopy, *SCL7A11* solute carrier family 7 member 11, *GPX4* glutathione peroxidase 4, *GAPDH* glyceraldehyde-3-phosphate dehydrogenase

We subsequently performed further studies to validate oxidative stress damage and ferroptosis. Compared with those in the TBHP and TBHP + Era group, ROS levels in the TBHP + Era + Mel group were reduced by half (Fig. 6a), increased intracellular Fe and Fe^2+^ contents (Fig. 6b), reduced SOD activity (Fig. 6c), increased MDA levels (Fig. 6d), and the inhibition of the glutathione reductase system alongside elevated oxidase activity (Fig. 6e) in the TBHP + Era group were alleviated by melatonin treatment, which indicated that the influence of melatonin on HUVECs was correlated with the inhibition of ferroptosis. TEM analysis revealed normal mitochondrial structure in the TBHP + Mel and TBHP + Era + Mel groups (Fig. 6f). Moreover, we assayed the accumulation of ROS using a C11 BODIPY probe, as the probe bound to lipid peroxides exhibited green fluorescence [38]. As shown in Figure 6g and h, lipid peroxidation expression was noticeably downregulated in the TBHP + Era + Mel group. The attenuation of intracellular ferrous ion accumulation was also observed in the TBHP + Era + Mel group (Fig. 6i and j). Finally, the protein expression of the ferroptosis markers SCL7A11 and GPX4 was measured. The data revealed that melatonin reversed the reduction in the expression of ferroprotective proteins caused by TBHP and erastin in HUVECs (Fig. 6k).

### Nrf2/HO-1 signaling is involved in melatonin-mediated ferroptosis in skin flaps and *tert*-butyl hydroperoxide– induced injury in human umbilical vein endothelial cells

**Figure 7:**
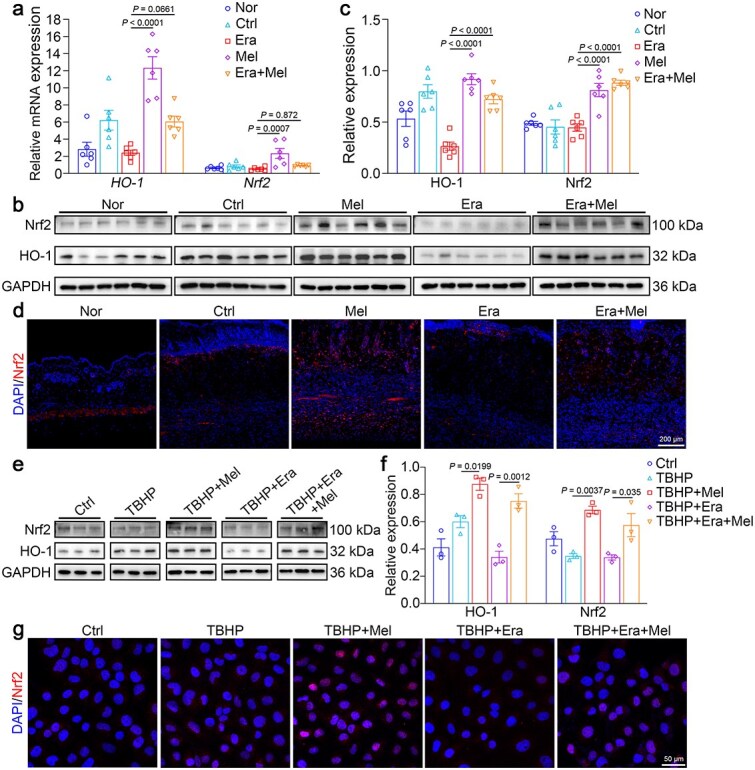
Nrf2/HO-1 signaling is involved in melatonin-mediated ferroptosis. (**a**) qRT-PCR analysis of *Nrf2* and *HO-1* mRNA expression in random skin flaps from mice (*n* = 6 per group). (**b**, **c**) Western blot and quantitative analysis of Nrf2 and HO-1 expression in mouse flap tissues. (**d**) Representative immunofluorescence staining for Nrf2 (red) in flaps treated with saline, melatonin, Era + melatonin, and normal skin. Scale bar: 200 μm. (**e**, **f**) Western blot and quantitative analysis of Nrf2 and HO-1 expression in endothelial cells (*n* = 3 per group). (**g**) Representative immunofluorescence staining of Nrf2 (red) in HUVECs. Scale bar: 50 μm.The data are presented as the means ± SEM, one-way ANOVA (a, c, f) with Tukey’s multiple comparisons test. *Nor* normal, Ctrl control, *Era* erastin, *Mel* melatonin, *TBHP tert*-butyl hydroperoxide, *HO-1* heme oxygenase-1, *Nrf2* nuclear factor E2-related factor 2, *GAPDH* glyceraldehyde-3-phosphate dehydrogenase

Currently, the Nrf2/HO-1 signaling pathway is recognized as crucial for the regulation of ferroptosis [25, 39]. Nrf2 acts as a master transcriptional factor for the cellular antioxidant and is a well-established upstream activator of both *SLC7A11* and *GPX4* gene expression. Furthermore, HO-1, a major downstream target of Nrf2, has been reported to exert antiferroptotic effects [40]. To explore melatonin’s antiferroptotic mechanisms, we examined the Nrf2/HO-1 signaling pathway. Melatonin treatment increased the mRNA and protein levels of Nrf2 and HO-1 in mouse skin flap tissues (Fig. 7a–c). Immunofluorescence analysis further revealed increased Nrf2 expression and facilitated the nuclear translocation of Nrf2 following melatonin treatment (Fig. 7d). Consistent with these findings, melatonin elevated Nrf2 and HO-1 protein *in vitro* and facilitated Nrf2 nuclear translocation, indicating pathway activation (Fig. 7e–g). Hence, we hypothesized that melatonin could mediate the survival of ischemic flap by suppressing ferroptosis, which is involved with the stimulation of the Nrf2/HO-1 signaling pathway.

### Melatonin enhances the survival of nonhuman primate skin flaps by inhibiting ferroptosis

**Figure 8:**
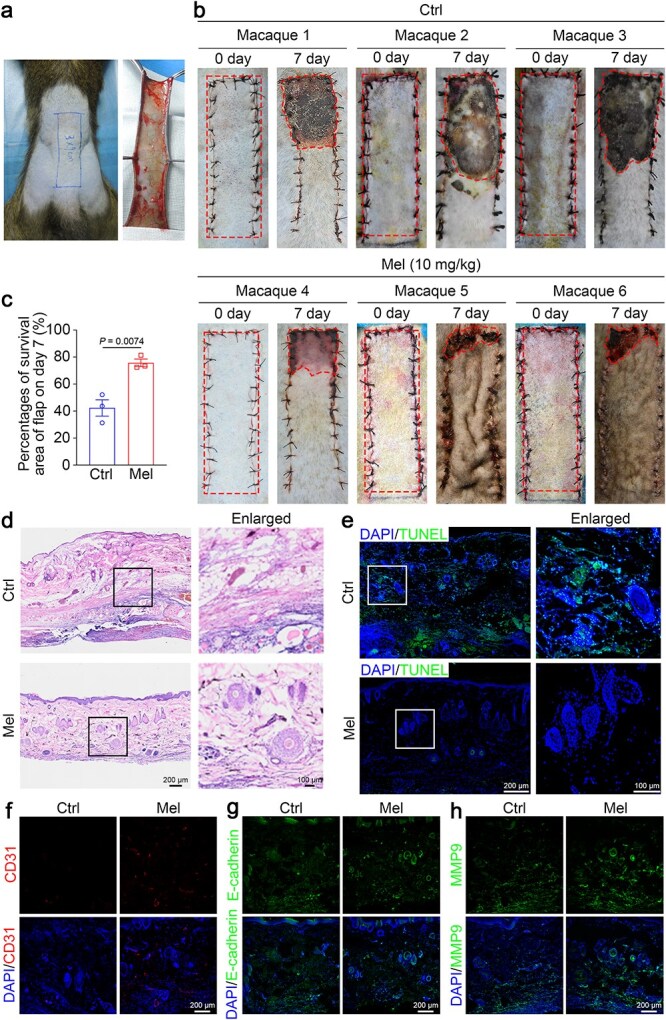
Melatonin promotes the survival of random skin flaps in macaques. A skin flap wound model was developed on the back of macaques. The macaques received exogenous melatonin treatment for seven continuous days, with the control group administered an equivalent dose of saline. (**a**) Schematic showing the design of a 3 × 9 cm flap based on the anterior superior iliac spine on the dorsum of each macaque. (**b**) Gross appearance of flaps on days 0, 3, and 7 after treatment with saline or melatonin. (**c**) Quantification of the survival area on day 7 (*n* = 3 per group). (**d**) Representative H&E-stained images of saline- or melatonin-treated flaps. (**e**) Representative images of TUNEL staining indicating cell death in macaque skin tissues treated with saline or melatonin. Scale bar: 200 μm; 100 μm. (**f**–**h**) Representative fluorescence immunostaining for CD31 (red), E-cadherin (green), and MMP9 (green) within macaque flaps. Scale bar: 200 μm. The data are presented as the means ± SEM, and Student’s *t*-tests were performed for (c) comparisons. *Ctrl* control, *Mel* melatonin, *TUNEL* terminal dUTP nick end labeling, *MMP9* matrix metallopeptidase 9

NHPs exhibit similarities to humans in anatomy, physiology, metabolism, and endocrinology. Thus, NHPs serve as an appropriate model for evaluating flap therapy and ultimately translate preclinical research into practical clinical practices [41–43]. A previous study has established that ferroptosis is involved in the melatonin-mediated healing of skin flaps. In this study, we assessed the efficacy and safety of melatonin by establishing a random skin flap model in cynomolgus monkeys. Six healthy adult macaques were employed in the study. Following the creation of a 3 cm × 9 cm skin flap on the dorsal midline, the macaques were randomly divided into two groups (Fig. 8a). Macaques were orally administered melatonin (10 mg/kg) immediately after surgery for 7 days, after which appearance, color, and necrosis of the flaps were observed.

Routine counts and biochemical analyses were first conducted at the indicated times to assess the safety of melatonin, and the data indicated that compared with those before surgery, none of the values after treatment varied (Table S2). Area II skin flaps were harvested and analyzed on day 7. We first examined the morphological and histological changes in the flap. Melatonin-treated skin flaps exhibited markedly improved necrosis, which was manifested primarily as reduced swelling and reduced necrotic areas, and the survival of the flaps was markedly enhanced (Fig. 8b and c). In contrast, the control group exhibited widespread flap necrosis (over 50%) (Fig. 8b and c). H&E staining revealed that melatonin promoted neovascularization of the skin in macaques while inhibiting skin edema and inflammation (Fig. 8d). Consistent with these findings, TUNEL staining revealed a marked reduction in TUNEL-positive cells in melatonin-treated flaps compared with controls (Fig. 8e), indicating attenuated tissue injury and cell death. High expression of angiogenesis-related factors (CD31, E-cadherin, and MMP9) were detected using immunofluorescence (Fig. 8f–h).

**Figure 9:**
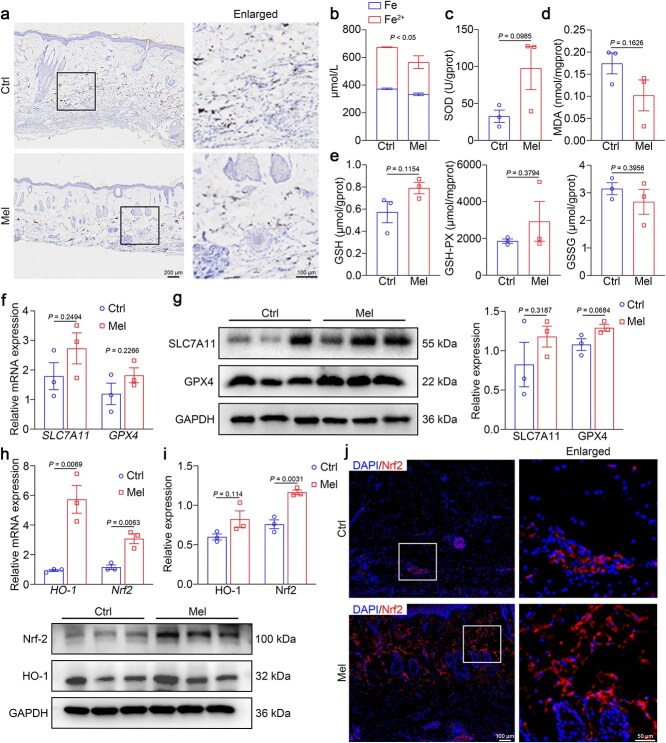
Melatonin inhibits ferroptosis in macaques. (**a**) Representative images of Prussian blue staining in monkey flap tissues treated with saline or melatonin. Scale bar: 200 μm; 100 μm. (**b**) Quantification of Fe and Fe^2+^ levels in flap tissues. (**c**–**e**) Quantification of the endogenous antioxidants SOD, MDA, GSH, GSH-PX, and GSSG in flap tissues. (**f**) Analysis of *SLC7A11* and *GPX4* mRNA expression using qRT-PCR. (**g**) SLC7A11 and GPX4 protein expression analyzed using western blot in flap tissues. (**h**, **i**) Nrf2 and HO-1 expression analyzed using western blot in macaque flap tissues (*n* = 3 per group). (**j**) Representative immunofluorescence staining for Nrf2 (red) in macaque flap tissue. Scale bar: 100 μm; 50 μm. The data are presented as the means ± SEM, and Student’s *t*-tests were performed for (b–i) comparisons. *Ctrl* control, *Mel* melatonin, *SOD* superoxide dismutase, *MDA* malondialdehyde, *GSH* glutathione, *GSH-PX* glutathione peroxidase, *GSSG* oxidized glutathione, *SCL7A11* solute carrier family 7 member 11, *GPX4* glutathione peroxidase 4, *Nrf2* nuclear factor E2-related factor 2, *HO-1* heme oxygenase-1, *GAPDH* glyceraldehyde-3-phosphate dehydrogenase

Next, we investigated whether melatonin could promote flap survival by inhibiting ferroptosis. Ferroptosis was suppressed in the melatonin group, as indicated by a reduction in iron accumulation (Fig. 9a), a decrease in Fe^2+^ (Fig. 9b), an increase in SOD activity (Fig. 9c), inhibition of MDA activity (Fig. 9d), an increase in GSH and GSH-PX contents (Fig. 9e), and depletion of GSSH (Fig. 9e). More importantly, Consistent with previous findings, we also detected increased expression of ferroptosis-protective genes (SLC7A11 and GPX4) (Fig. 9f and g). We further observed upregulated Nrf2 and heme HO-1 expression in melatonin-treated macaque skin flaps, along with the nuclear translocation of Nrf2 (Fig. 9h–j). In short, our results indicated that melatonin facilitated the healing of distal ischemic skin flaps in NHPs by inhibiting ferroptosis.

## Discussion

Random skin flaps represent among the most frequently utilized techniques in reconstructive surgery and are widely employed clinically for repairing various forms of tissue damage [44]. However, their application is considerably limited by postoperative complications, particularly distal tissue ischemia and necrosis. Thus, there’s a pressing for new therapeutic strategies to increase flap survival [8, 44]. The main causes of distal tissue necrosis are inadequate blood supply and ischemia–reperfusion injury [7]. Numerous studies have aimed to improve flap viability by addressing these mechanisms [45, 46]. Although progress has been made in current research, clinical outcomes remain unsatisfactory.

Melatonin, a naturally occurring indole heterocyclic compound, is primarily synthesized and secreted by the pineal gland in the brain [12]. Beyond its well-known chronobiological functions, melatonin has emerged as a potent endogenous antioxidant, offering protection against oxidative stress and inflammation [14, 47]. Research has consistently demonstrated its therapeutic potential in humans, highlighting its ability to mitigate oxidative damage and reduce inflammation [15, 48]. In models of ischemia–reperfusion injury, melatonin has been shown to preserve mitochondrial function, minimizing organ damage and promoting cellular recovery [49]. Furthermore, studies have demonstrated that melatonin enhances angiogenesis, a critical process for improving the survival of perforator flaps by ensuring adequate blood supply [46, 50]. Building on these studies, we hypothesized that melatonin would increase flap survival by facilitating angiogenesis and attenuating oxidative stress. To test this hypothesis, exogenous melatonin was orally administered to mice and macaques with random flap models. The results demonstrated that melatonin treatment significantly reduced flap necrosis, and enhanced blood perfusion following angiogenesis plays a pivotal step in this repair process. Immunofluorescence assays revealed marked upregulation of CD31 signaling following melatonin administration. Importantly, the colocalization of CD31 with macrophage markers (F4/80 and CD206) was minimal, suggesting that the increased CD31 expression was primarily derived from endothelial cells rather than macrophage. The results indicated that melatonin directly promotes endothelial angiogenesis. Furthermore, melatonin treatment upregulated the expression of MMP9, a key angiogenesis-promoting factor and E-cadherin, a crucial cell adhesion molecule, which are involved in tissue repair [30, 31, 50]. These results collectively indicated that melatonin facilitated vascular regeneration and supports structural recovery in ischemic flap tissues.

Oxidative stress plays a key role in ischemia–reperfusion injury [17]. TBHP primarily promotes ferroptosis through oxidative stress–driven lipid peroxidation, whereas erastin induces ferroptosis by disrupting the Xc^−^/GSH/GPX4 axis [25, 26]. The consistent protective effects of melatonin observed under both conditions indicate its antiferroptotic action is not limited to a single upstream pathway. Instead, melatonin seems to broadly reduce ferroptotic injury by maintaining redox balance and limiting lipid peroxidation, which enhanced endothelial cell survival and flap viability. Since endothelial cell growth and migration are crucial for angiogenesis, enhanced cell functions and tube formation collectively demonstrated that exogenous melatonin boosts flap survival through enhancing neovascularization and reducing oxidative stress [51].

During tissue repair after injury, ischemic damage is often unavoidable, primarily due to the onset of oxidative stress [52]. Under such conditions, tissues produce large amount of superoxide anions, which damage membrane phospholipids and lead to cell death [52, 53]. These superoxide anions also break down the extracellular matrix, exacerbating tissue necrosis [54]. Previous studies have shown that suppression ferroptosis can reduce oxidative damage, and melatonin has been found to lower ferroptosis levels in various disease [24, 55]. We hypothesized that melatonin may improve flap tissue survival by reducing oxidative stress through ferroptosis inhibition. Hence, we assessed ferroptosis in mouse flap tissues and TBHP-HUVEC model. Iron accumulation in the melatonin-treated group was notably lower than the control group. Western blot results revealed that melatonin markedly upregulated the content of SLC7A11 and GPX4, two key ferroptosis markers [21, 56]. Mitochondrial morphology in the melatonin-treated group remained intact, without shrinkage and condensation compared with those in the control group. Given the observed mitochondrial protection conferred by melatonin, it is plausible that dihydroorotate dehydrogenase (DHODH)—associated pathways might also participate in limiting ferroptotic damage, although this possibility requires further investigation [57, 58]. We also measured oxidative damage by assessing MDA contents (a product of lipid peroxidation and reflects the severity) and SOD activity (an antioxidant enzyme that scavenges superoxide anions) [35, 36]. Our findings showed that melatonin markedly reduced MDA concentrations and enhanced SOD activity, suggesting a protective effect against oxidative stress. These results supported the conclusion that melatonin attenuated oxidative stress and enhanced flap survival by inhibiting ferroptosis. Finally, we explored the possible mechanisms of how melatonin inhibited ferroptosis. The Nrf2/HO-1 pathway is crucial for regulating oxidative stress and ferroptosis [39]. We observed increased Nrf2 and HO-1 expression in skin flap tissues and endothelial cells, along with increased nuclear translocation of Nrf2, suggesting that melatonin potentially inhibited ferroptosis by activating this pathway.

NHP models are crucial for translating experimental findings into clinical practice [42]. Building on our prior work with murine models and aiming to overcome the limitations of random skin flaps in clinical applications, we conducted experiments using NHP models. Six cynomolgus monkeys subjected to random flap surgery, and three of them were received exogenous melatonin. The outcomes showed a notable improvement in flap in the melatonin-treated. Similar to mice studies, melatonin attenuated oxidative stress by suppressing ferroptosis and stimulating the Nrf2/HO-1 pathway in the flap tissue, validating its role in boosting flap viability. Hematological analysis revealed no pathological alterations or adverse effects, supporting the safety and feasibility of melatonin administration.

## Conclusions

This study revealed that melatonin notably protects against ischemic flap necrosis in both rodents and NHPs. The findings revealed that melatonin’s therapeutic effect was primarily through its ability to alleviate oxidative stress, reduce tissue necrosis, and promote angiogenesis by inhibiting ferroptosis. Additionally, these mechanisms are seemingly associated with the activation of the Nrf2/HO-1 signaling pathway. In summary, these findings suggest that melatonin represents a highly promising and translatable therapeutic agent, offering a novel and effective strategy for enhancing the survival rate of flaps in plastic surgery.

## Supplementary Material

Supplementary_material_clean_tkag012

## Data Availability

All the data generated for this study can be obtained from the corresponding author.
