# Supplementary material for: Melatonin promotes skin flap survival by inhibiting ferroptosis via activation of the Nrf2/HO-1 pathway
Source: Burns Trauma. 2026 Feb 2;14:tkag012. doi: 10.1093/burnst/tkag012 (PMC13137327; doi:10.1093/burnst/tkag012)
Supplement: Supplementary_material_clean_tkag012 [file supplementary_material_clean_tkag012.docx]

**Figure S1**

**
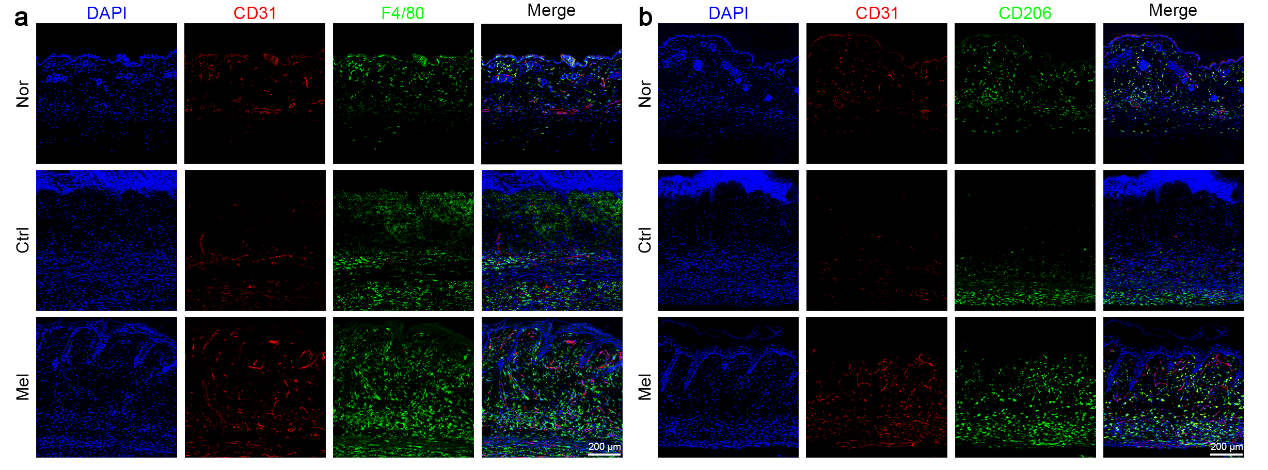
**

**Figure S1.** (**a**) Representative images of co-staining of CD31 (red) with F4/80 (macrophage marker, green) in mouse skin flap tissue treated with normal, saline or melatonin. (**b**) Representative images of co-staining of CD31 (red) with CD206 (M2 macrophage marker, green) in mouse skin flap tissue treated with normal, saline or melatonin.

**Table S1. Routine blood and biochemical analysis of macaques** **(macaques 1-6) at various time points after random skin flap surgery.**

|  |  | **Routine blood test** | | | | | | |  | **Blood biochemistry test** | | | | |
| --- | --- | --- | --- | --- | --- | --- | --- | --- | --- | --- | --- | --- | --- | --- |
|  | Days | WBC  (10^9/L) | RBC  (10^12/L) | PLT  (10^9/L) | HGB  (g/L) | NEU  (10^9/L) | LYM  (10^9/L) | MONO  (10^9/L) |  | ALT  (U/L) | AST  (U/L) | TBIL  (μmol/L) | UREA  (mmol/L) | CREA  (μmol/L) |
| Macaque 1 | Pre-surgery | 6.03 | 4.52 | 543.0 | 114.0 | 2.14 | 3.11 | 0.37 |  | 51.0 | 107.8 | 1.43 | 7.43 | 32.8 |
|  | 0 day  (Surgery) | 6.56 | 4.54 | 518.0 | 116.0 | 2.28 | 3.44 | 0.38 |  | 49.9 | 108.8 | 1.59 | 7.37 | 31.7 |
|  | 1 day | 6.23 | 4.64 | 521.0 | 115.0 | 2.18 | 3.25 | 0.41 |  | 0.1 | 108.8 | 1.43 | 7.27 | 33.1 |
|  | 3 day | 7.61 | 4.96 | 389.0 | 129.0 | 2.45 | 4.10 | 0.47 |  | 50.9 | 30.5 | 1.40 | 6.92 | 43.3 |
|  | 7 day | 7.32 | 4.91 | 416.0 | 131.0 | 2.33 | 3.84 | 0.53 |  | 50.1 | 30.5 | 1.49 | 7.20 | 43.3 |
|  | 14 day | 5.15 | 3.97 | 430.0 | 101.0 | 3.60 | 1.25 | 0.14 |  | 59.4 | 130.6 | 2.07 | 6.58 | 22.9 |
| Macaque 2 | Pre-surgery | 10.34 | 3.48 | 722.0 | 86.0 | 7.44 | 2.03 | 0.79 |  | 29.5 | 27.4 | 1.49 | 5.70 | 66.6 |
|  | 0 day  (Surgery) | 10.70 | 3.47 | 720.0 | 87.0 | 8.21 | 1.78 | 0.63 |  | 42.4 | 48.1 | 1.98 | 7.29 | 75.7 |
|  | 1 day | 10.80 | 3.42 | 721.0 | 87.0 | 7.86 | 2.05 | 0.78 |  | 42.4 | 48.5 | 1.82 | 7.14 | 74.6 |
|  | 3 day | 12.56 | 5.34 | 475.0 | 133.0 | 5.12 | 6.23 | 0.85 |  | 30.3 | 26.7 | 2.33 | 3.38 | 44.8 |
|  | 7 day | 12.52 | 5.32 | 457.0 | 132.0 | 5.18 | 6.27 | 0.71 |  | 30.3 | 26.7 | 2.18 | 3.34 | 46.6 |
|  | 14 day | 13.27 | 4.65 | 438.0 | 114.0 | 9.49 | 2.89 | 0.48 |  | 59.7 | 143.4 | 2.15 | 6.66 | 22.9 |
| Macaque 3 | Pre-surgery | 8.77 | 5.53 | 516.0 | 123.0 | 3.98 | 4.20 | 0.47 |  | 28.7 | 26.3 | 1.32 | 5.66 | 67.3 |
|  | 0 day  (Surgery) | 8.61 | 5.57 | 511.0 | 121.0 | 3.94 | 3.93 | 0.66 |  | 103.6 | 103.6 | 1.57 | 6.51 | 39.6 |
|  | 1 day | 6.39 | 5.31 | 493.0 | 136.0 | 1.50 | 4.09 | 0.36 |  | 37.7 | 34.1 | 1.35 | 6.83 | 81.2 |
|  | 3 day | 6.63 | 5.29 | 488.0 | 133.0 | 1.35 | 4.27 | 0.32 |  | 38.4 | 34.1 | 1.59 | 6.79 | 80.5 |
|  | 7 day | 6.86 | 5.20 | 471.0 | 135.0 | 1.53 | 4.49 | 0.36 |  | 24.4 | 67.1 | 2.40 | 6.26 | 71.8 |
|  | 14 day | 5.49 | 3.97 | 457.0 | 104.0 | 3.92 | 1.26 | 0.12 |  | 52.4 | 109.9 | 1.51 | 7.48 | 32.4 |
| Macaque 4 | Pre-operation | 10.05 | 5.09 | 418.0 | 108.0 | 4.69 | 4.63 | 0.58 |  | 27.5 | 39.0 | 1.67 | 5.01 | 60.8 |
|  | 0 day  (Surgery) | 10.74 | 5.06 | 370.0 | 111.0 | 4.94 | 5.20 | 0.43 |  | 32.4 | 32.7 | 1.74 | 3.25 | 48.0 |
|  | 1 day | 6.23 | 5.22 | 481.0 | 133.0 | 1.48 | 4.01 | 0.32 |  | 32.8 | 31.2 | 1.66 | 3.41 | 49.4 |
|  | 3 day | 6.15 | 5.23 | 480.0 | 132.0 | 1.42 | 4.00 | 0.33 |  | 66.3 | 43.2 | 2.31 | 3.95 | 48.4 |
|  | 7 day | 6.79 | 5.21 | 468.0 | 135.0 | 1.57 | 4.41 | 0.35 |  | 66.3 | 43.9 | 2.15 | 4.02 | 49.1 |
|  | 14 day | 13.00 | 4.63 | 419.0 | 114.0 | 9.13 | 2.98 | 0.48 |  | 29.2 | 29.5 | 2.18 | 3.40 | 45.9 |
| Macaque 5 | Pre-surgery | 14.14 | 3.42 | 585.0 | 75.0 | 11.78 | 1.26 | 0.99 |  | 30.0 | 28.0 | 1.50 | 7.0 | 61.0 |
|  | 0 day  (Surgery) | 9.52 | 5.03 | 385.0 | 107.0 | 4.63 | 4.31 | 0.48 |  | 33.0 | 28.0 | ＜3 | 5.3 | 75.1 |
|  | 1 day | 14.39 | 3.33 | 562.0 | 75.0 | 12.34 | 1.25 | 0.72 |  | 33.0 | 29.0 | ＜3 | 6.7 | 84.0 |
|  | 3 day | 5.67 | 3.72 | 359.0 | 80.0 | 4.36 | 1.11 | 0.18 |  | 35.0 | 28.0 | ＜3 | 6.8 | 75.4 |
|  | 7 day | 5.77 | 3.83 | 396.0 | 81.0 | 4.21 | 1.26 | 0.28 |  | 32.0 | 29.0 | ＜3 | 6.9 | 73.5 |
|  | 14 day | 5.09 | 4.54 | 452.0 | 122.0 | 2.06 | 2.29 | 0.38 |  | 30.7 | 29.5 | ＜3 | 3.40 | 46.2 |
| Macaque 6 | Pre-surgery | 10.08 | 4.46 | 555.0 | 108.0 | 4.85 | 4.32 | 0.49 |  | 46.0 | 43.0 | ＜3 | 5.2 | 46.7 |
|  | 0 day  (Surgery) | 6.67 | 4.87 | 408.0 | 126.0 | 1.93 | 3.82 | 0.38 |  | 44.0 | 41.0 | ＜3 | 5.2 | 57.0 |
|  | 1 day | 2.61 | 3.58 | 269.0 | 75.0 | 1.81 | 0.63 | 0.13 |  | 45.0 | 43.0 | ＜3 | 5.1 | 50.1 |
|  | 3 day | 2.80 | 3.21 | 301.0 | 70.0 | 2.02 | 0.67 | 0.07 |  | 46.0 | 42.0 | ＜3 | 5.0 | 45.3 |
|  | 7 day | 14.37 | 3.45 | 587.0 | 77.0 | 11.89 | 1.26 | 1.14 |  | 43.0 | 40.0 | ＜3 | 4.6 | 46.9 |
|  | 14 day | 4.88 | 4.45 | 433.0 | 114.0 | 2.08 | 2.22 | 0.25 |  | 30.3 | 27.0 | ＜3 | 3.40 | 45.2 |

Red text indicates the beginning of melatonin injection. WBC, white blood cell; RBC, red blood cell; HGB, hemoglobin; HCT: hematocrit; PLT: platelet; NEU, neutrophil; LYM, lymphocyte; MONO, monocyte; ALT: alanine aminotransferase; AST, aspartate aminotransferase; TBIL, total bilirubin; CREA, creatinine

**Table S2. Primer sequence list for qRT-PCR**

| **Gene** | **Forward Primer**  **(5’-3’)** | **Reverse Primer**  **(5’-3’)** |
| --- | --- | --- |
| *Musculus* |  |  |
| *SLC7A11* | CCTCTGCCAGCTGTTATTGTT | CCTGGCAAAACTGAGGAAAT |
| *GPX4* | ATGAAAGTCCAGCCCAAGG | CGGCAGGTCCTTCTCTATCA |
| *HO-1* | AACTTTCAGAAGGGTCAGGTGTC | CTCCTCAGGGAAGTAGAGTGGG |
| *Nrf2* | TGTCTTAATACCGAAAACAAGCAGC | GACCACAGTTGCCCACTTCTTTT |
| *β-actin* | GACCTCTATGCCAACACAGTGC | GTACTCCTGCTTGCTGATCCAC |
| *Macaque* |  |  |
| *SLC7A11* | ACTCCTCATAATACGCCCTGC | AGCCCACAGCTGTAATGAGC |
| *GPX4* | GGTACCATGTGCGCGT | TCTTTCGCGGAAAACTCGTG |
| *HO-1* | CTCCTCTCGAGCGTCCTCAG | ATCCTGGGGCATGCTGTCG |
| *Nrf2* | AGCTCAGCATGATGGAGTTGG | CTCATACTCTTTCCGTCGCTG |
| *β-actin* | CCTTCCTTCCTGGGCATGG | ACGGATGTCCACGTCACAC |

SCL7A11, solute carrier family 7 member 11; GPX4, glutathione peroxidase 4; HO-1, Heme oxygenase-1; Nrf2, nuclear factor E2-related factor 2
